# A Complex Journey: Cell Wall Remodeling, Interactions, and Integrity During Pollen Tube Growth

**DOI:** 10.3389/fpls.2020.599247

**Published:** 2020-11-30

**Authors:** Milagros Cascallares, Nicolás Setzes, Fernanda Marchetti, Gabriel Alejandro López, Ayelén Mariana Distéfano, Maximiliano Cainzos, Eduardo Zabaleta, Gabriela Carolina Pagnussat

**Affiliations:** Instituto de Investigaciones Biológicas, Universidad Nacional de Mar del Plata, CONICET, Mar del Plata, Argentina

**Keywords:** pollen, cell wall, plant fertilization, cell wall remodeling, pollen tube

## Abstract

In flowering plants, pollen tubes undergo a journey that starts in the stigma and ends in the ovule with the delivery of the sperm cells to achieve double fertilization. The pollen cell wall plays an essential role to accomplish all the steps required for the successful delivery of the male gametes. This extended path involves female tissue recognition, rapid hydration and germination, polar growth, and a tight regulation of cell wall synthesis and modification, as its properties change not only along the pollen tube but also in response to guidance cues inside the pistil. In this review, we focus on the most recent advances in elucidating the molecular mechanisms involved in the regulation of cell wall synthesis and modification during pollen germination, pollen tube growth, and rupture.

## Introduction: The Pollen Cell Wall

Angiosperms or flowering plants produce two types of spores. During megasporogenesis, diploid megaspore mother cells undergo meiosis, giving rise to haploid megaspores. A functional megaspore develops into a female gametophyte in a process called megagametogenesis. Conversely, during microsporogenesis, diploid microspore mother cells give rise to microspores, which then undergo microgametogenesis to develop male gametophytes.

The male gametophyte or pollen grain (PG) develops in specialized organs called anthers, which are located atop filaments around the carpel. The innermost layer of the anther locules is the tapetum, a tissue that provides nutrients and precursors for PG development and pollen wall formation. The pollen performs a critical step in the plant life cycle by transporting the male gametes to the ovule for fertilization. Depending on the species, this journey can be long and might include exposure to a wide range of abiotic stresses from which PGs are protected by a unique and specialized cell wall (CW; Edlund et al., 2004; Lou et al., 2014).

The pollen CW was a key innovation that enabled plant life on land. It is a sophisticated multi-layered structure basically composed by an outer sporophyte-derived exine and an inner gametophyte-derived intine layer (Ariizumi and Toriyama, 2011). The intine layer resembles the primary CW of somatic plant cells and consists of cellulose, hemicellulose, and pectin together with CW-associated proteins. The exine layer creates distinctive pollen wall patterns that are conserved within species and serves as a robust barrier for protection while allowing pollen tube (PT) emergence and pollen-stigma recognition (Radja et al., 2019). These patterns on the pollen surface contain layers known as sexine and nexine. While nexine includes arabinogalactan proteins (AGPs) generally rich in hydroxyproline (Jia et al., 2015), the major constituent of sexine is sporopollenin, a resistant biopolymer made of polyhydroxylated aliphatic chains and aromatic rings (Ariizumi and Toriyama, 2011; Xiong et al., 2020).

**Figure 1:**
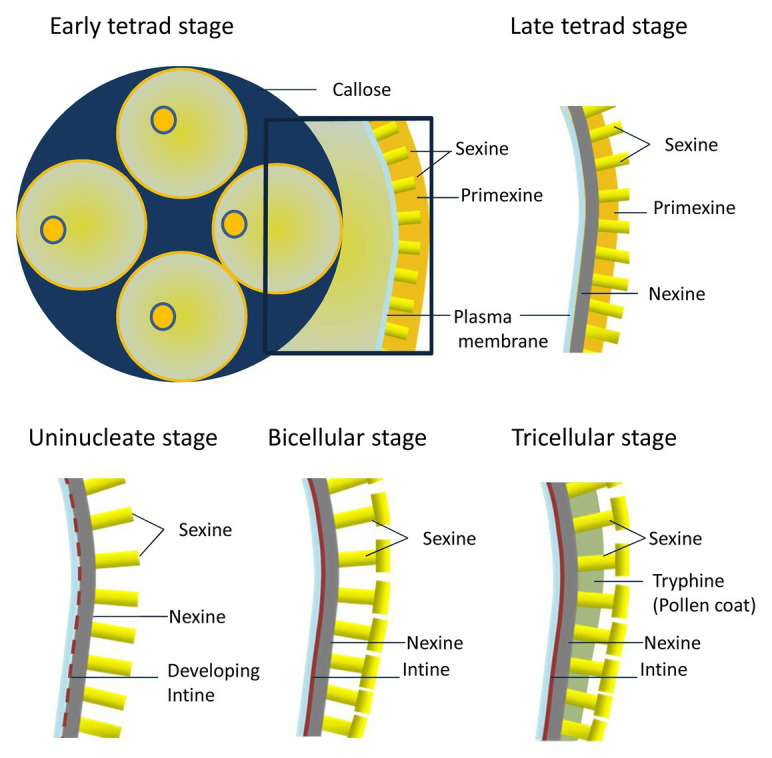
Diagram showing pollen cell wall deposition and patterning.

All these pollen wall components are precisely deposited at specific developmental stages during microgametogenesis (Figure 1; Heslop-Harrison, 1968; Xu et al., 2016). Right after meiosis, at the early tetrad stage, the callose that surrounds the microspores is degraded by callases secreted from the tapetum. Following callose degradation, the developing microspores form primexine, a microfibrillar matrix mainly consisting of cellulose. Primexin functions as a template for the deposition of sporopollenin precursors, which are provided by the tapetum (Shi et al., 2015). At the late tetrad stage, nexine starts to gradually accumulate in the primexine matrix beneath sexine. At the uninucleate-free microspore stage, pollen wall development is characterized by rapid sporopollenin deposition and the formation of a patterned exine structure (Figure 1). After microspore expansion, intine forms below the nexine (Li et al., 2017a). At late stages of pollen development, the tapetum undergoes programmed cell death (PCD) which results in a mixture of protein and wax, known as tryphine, deposited on the surface of the exine (Figure 1; Rejón et al., 2016). All these structural components allow the pollen to resist environmental stresses, such as desiccation, UV radiation, and microbial attack as well as to allow specific adhesion to the stigma. Mature PGs undergo a desiccation process before being released from the anther. Once the PGs make contact with the stigma, they rehydrate, allowing them to become metabolically active (Edlund et al., 2004; Kim et al., 2019; Bosch and Wang, 2020). The dehydrated PG contains all the proteins required for the key steps that will take place once the PG lands on the stigma, such as pollen hydration, germination, and initial tube growth (Mayank et al., 2012).

## Landing on the Stigma: Pollen Recognition, Hydration, and Germination

**Figure 2:**
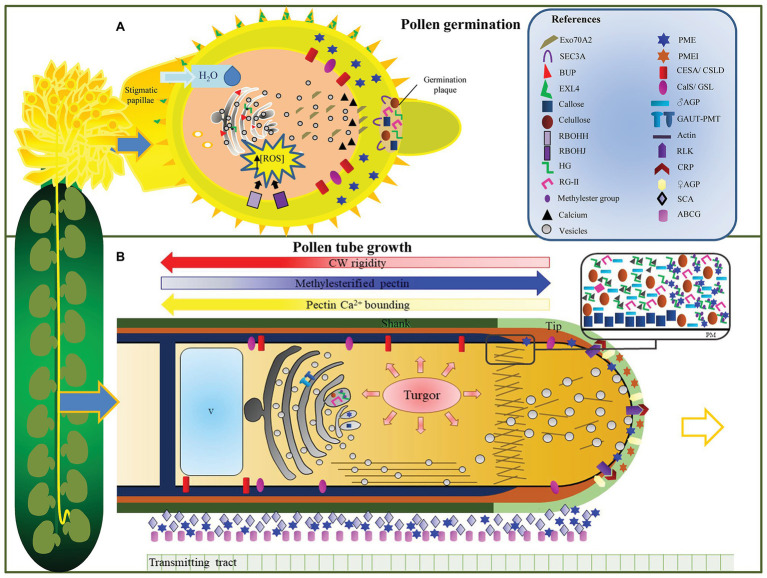
The pollen cell wall plays a central role in guaranteeing the delivery of the male gametes. In these cartoons, we summarize the changes and regulatory mechanisms underlying cell wall deposition and modifications that occur during pollen grain germination **(A)** and pollen tube (PT) elongation **(B)**. **(A)** Pollen grain germination. Early events include extensive cell wall (CW) material trafficking from the Golgi apparatus to the PT emergence site, while other CW components are synthesized in the plasma membrane. Water uptake from the stigmatic papillae is facilitated by pectin methylesterases’ (PMEs) activity. PMEs are needed for homogalacturonan demethylesterification, increasing the hydrophilic properties of the CW. The alteration of the mechanical properties of the CW that is observed prior to germination relies on the accumulation of reactive oxygen species inside the pollen grain. An intine-like germination plaque is established at the germination site, which is composed of cellulose, callose, pectin, and at least partially de-esterified pectin. **(B)** PT elongation and guidance through the transmitting tract (TT). This model shows the polysaccharides that compose the pollen tube CW, which varies along its extension. The main players in cell wall remodeling are also shown. Turgor pressure drives PT growth, while Golgi-derived vesicles are released in the apical zone where they fuse to deliver their polysaccharides and/or remodeling proteins. Male receptor-like-kinase complexes interact with synergid cysteine-rich proteins, guiding PT growth towards the ovule. Arabinogalactan proteins from the pistil TT are also shown, along with other proteins (stigma/stylar cysteine-rich adhesin protein and ATP binding cassette subfamily G) that facilitate the adhesion and elongation of PT inside the pistil.

Once landed on the stigma, PGs need to adhere and hydrate before germination is initiated (Dresselhaus and Franklin-Tong, 2013; Bosch and Wang, 2020; Figure 2A). The first stage of male-female recognition occurs when the PGs make the first contact with the stigma (Dresselhaus and Franklin-Tong, 2013; Bosch and Wang, 2020). This interaction involves a series of complex and cooperative processes between the PGs and the receptive pistil that are crucial for successful fertilization (Chapman and Goring, 2010; Rozier et al., 2020). The chain of events that follows may happen on a wet or a dry stigma, depending on the plant species (Doucet et al., 2016). Wet stigmas are thought to be less specific than dry stigmas because they produce superficial secretions that sometimes allow the development of pollen from other species and even spores from plant pathogens (Lush et al., 1998; Wolters-Arts et al., 1998; Quiapim et al., 2009; Sang et al., 2012). On the other hand, the absence of such secretions provides dry stigmas with full control over the initial contact with the pollen (Dickinson, 1995; Samuel et al., 2009; Chapman and Goring, 2010; Dresselhaus and Franklin-Tong, 2013; Goring, 2018; Bosch and Wang, 2020). Dry stigmas are able to accept compatible pollen for fertilization and to reject self-incompatible pollen while ignoring pollen from other species (Doucet et al., 2016; Goring, 2018). In Brassicaceae, once compatible PGs are recognized, water is transferred from the stigma to the PGs, which allows them to become metabolically active (Goring, 2018). This process involves EXO70A1, a putative component of the exocyst complex known to regulate polarized secretion that has been identified as a major component of the pollen—pistil interacting network. It is proposed that the exocyst docks vesicles at the plasma membrane just under the pollen contact site, which then release their content. Promoting pollen hydration (Samuel et al., 2009). While *EXO70A1* RNAi lines present a severe delay in PG hydration, overexpression of *EXO70A1* results in increased PG hydration (Samuel et al., 2009). Interestingly, a recent study found that phosphorylation of EXO70A1 by the MAPK3 and MAPK4 kinases is required for pollen germination and PT growth (Jamshed et al., 2020). Extracellular proteins are also required for PG hydration. For instance, the extracellular lipase EXL4 was shown to be necessary for efficient hydration, as *exl4-1* mutant PGs presented slow hydration and reduced esterase activity (Updegraff et al., 2009). CW-associated pollen-specific pectin methylesterases (PMEs) are also of key importance for this process. Homozygous *pme48* mutants show a strong delay in PG imbibition and germination. These defects have been associated with PME48 activity removing hydrophobic methylester groups from homogalacturonan (HG), which is thought to enhance the hydrophilic properties of the CW (Leroux et al., 2015).

Together with PG hydration, a germination site is established, which involves a tight regulation of CW synthesis and modification (Li et al., 2017b). Hoedemaekers et al. (2015) described that PG germination requires the formation of a germination plaque at the site of the future PT emergence. This intine-like plaque contains cellulose, callose, pectin, and at least partially de-esterified pectin. Proper formation of the germination plaque requires the activity of the Golgi-localized glycosyltransferase BURSTING POLLEN (BUP). It is proposed that BUP might be involved in pectin synthesis or delivery, as mutant PGs impaired in BUP show abnormalities in both plaque formation and PT growth (Hoedemaekers et al., 2015). PME48 is also thought to be required for the proper establishment of a germination site. As described in Leroux et al. (2015), *pme48* homozygous mutant lines showed PGs with multiple emerging PTs due to the presence of excessive highly methylesterified pectin in their intine wall. Additionally, massive secretion seems crucial for the deposition of the germination plaque at the site of PT emergence, as CW materials, proteins, and membrane components are rapidly transported to the germination site *via* the vesicle trafficking system. SEC3A is a subunit of the exocyst protein complex implicated in the tethering of secretory vesicles to the plasma membrane (Heider and Munson, 2012). Mutations in the *SEC3A* gene led to a disruption in PG germination as well as to severe perturbations in the deposition of CW material. Additionally, SEC3A was found to localize at the site of the future PT emergence, which suggests that polar localization of SEC3A in the PG is essential for successful pollen germination (Li et al., 2017b). The activation of PG metabolism leads to cytoplasmatic reorganization and also to the activation of specific proteins necessary for PT emergence (Kandasamy et al., 1994; Kim et al., 2019; Bosch and Wang, 2020).

PT emergence requires mechanical modifications on the PG cell wall. Loosening of the intine occurs at the germination site. Conversely, the CW on the rest of the pollen surface remains strong enough to support this initial growth. It has been shown that reactive oxygen species (ROS) have a key role throughout these CW modifications to ensure germination (Smirnova et al., 2014). The hydroxyl radical is involved in the local intine loosening that occurs at the germination pore, while H_2_O_2_ is required for intine strengthening through a peroxidase-mediated oxidative coupling of feruloyl-polysaccharides on the rest of the PG surface (Smirnova et al., 2014). Together with CW synthesis, ROS-generating NADPH oxidases can also be delivered to the plasma membrane to support germination and PT growth. An analysis of a CRISPR-generated *exo70a2* mutant by Marković et al. (2019) has revealed the importance of EXO70A2 on PG maturation, germination, and tube growth and has added more evidence that highlights the importance of ROS in these processes. EXO70A2 is a pollen-expressed exocyst subunit closely related to EXO70A1 that is required for ROS accumulation in germinating PGs (Marković et al., 2019). Two pollen-specific NADPH oxidases (RBOHH and RBOHJ) are also essential for ROS accumulation at the pollen grain cell wall during germination and PT growth (Lassig et al., 2014; Kaya et al., 2015; Jimenez-Quesada et al., 2019).

## The Pollen Tube Journey: Interaction with the Transmitting Tract Tissues

After germination, PTs elongate in one direction through the maternal tissues, originating a highly specialized polar structure (Guan et al., 2013; Dehors et al., 2019; Zhang et al., 2020). This structure is also reflected in an extremely polarized intracellular organization (Figure 2B). The cytoplasm distal region is highly vacuolated, and it is separated from the streaming region by callose plugs. Consequently, the cytoplasm remains in the front portion of the tube regardless of the PT length (Franklin-Tong, 1999). The streaming region is subdivided into the shank region, which contains the two sperm cells and the vegetative nucleus, the sub-apical organelle rich-region (especially ER and Golgi), and the tip region which lacks granular organelles (Franklin-Tong, 1999; Guan et al., 2013). The tip region is also characterized by the presence of an abundant number of transport vesicles that form an inverted-cone-shape pattern. These vesicles reflect the active exo-endocytosis processes that imply CW deposition and recycling (Wang et al., 2006; Moscatelli et al., 2007; Bove et al., 2008; Idilli et al., 2013). The PT growth pattern requires a highly dynamic cytoskeleton that participates in regulating the polar organization of the PT cytoplasm and targeted exocytosis (Idilli et al., 2013; Hamant et al., 2019). At the molecular level, an ROP GTPase-dependent signaling network, ROS, and Ca^2+^ gradients are reported to coordinate tip growth in PTs *via* their inter-connection with cytoskeletal elements (Franklin-Tong, 1999; Gu and Nielsen, 2013; Guan et al., 2013; Lassig et al., 2014; Diao et al., 2018; Zhang et al., 2020).

The main driving force of PT growth is hydrostatic pressure (turgor), which is restricted by the ability of the CW to expand, so the rate of growth is determined by the equilibrium between these two forces (Parre and Geitmann, 2005b; Cosgrove, 2018). As the PT is one of the fastest-growing cells in flowering plants, CW deposition, composition, and remodeling have to be precisely coordinated in a space-time scale to adjust the CW mechanical properties, allowing polar growth but avoiding premature PT burst (Winship et al., 2010; Dehors et al., 2019; Tang et al., 2020). In concordance with this, the biochemical composition of the pollen tube CW changes along the longitudinal axis. In the pollen tube shank, the CW is rigid, allowing the pollen tubes to resist turgor pressure, but flexible enough to permit cell expansion (Vogler et al., 2013). The CW of the pollen tube shank consists of a primary CW composed of polysaccharides like pectins and cellulose and a secondary CW composed almost exclusively of callose. At the tip, where the CW is expected to be softer than the distal part, the CW consists of a primary CW that is mostly composed of pectins, which provides this zone with elastic properties that allow directional expansion (Parre and Geitmann, 2005b; Winship et al., 2010).

To reach an ovule, the growing tube must penetrate the stigma and elongate through the transmitting tract and along an ovule funiculus towards the micropyle. Only when the PT reaches the female gametophyte inside the ovule does it bursts to release the two sperm cells. This journey implicates complex mechanisms that assure PT integrity and growth, adhesion to transmitting tract cells, and molecular communication between the PT and the maternal and gametophytic tissues of the ovule (Lord, 2003; Johnson et al., 2019; Reimann et al., 2020).

During its journey through the transmitting tract, the PT needs to adhere tightly to the extracellular matrix. The presence of a stigma/stylar cysteine-rich adhesin protein, which is similar to plant lipid transfer proteins, has been shown to be essential for PT adhesion and growth in *Lilium longiflorum* styles (Mollet et al., 2000; Park et al., 2000). In *Arabidopsis*, the transmitting tract is formed through a programmed cell death process, which provides enough intercellular space for PT growth. Several proteins involved in transmitting tract PCD have been identified. Some examples include NO TRANSMITTING TRACT, which encodes a C2H2/C2HC zinc finger transcription factor, HECATE 1 (HEC1), HEC2, HEC3, HALF FILLED, and SPATULA (Crawford et al., 2007; Crawford and Yanofsky, 2011; Mizuta and Higashiyama, 2018).

Glucose has also been proposed as an important molecule regulating PT growth, which might be perceived differently along the transmitting tract through the formation of a gradient. Although an interesting hypothesis, there are still no direct evidences to support this idea (Rottmann et al., 2018). In addition, a recent work demonstrated that the ATP binding cassette subfamily G (ABCG) transporters ABCG1 and ABCG16 are expressed in pistil tissues and are involved in auxin distribution and flow in the pistil, positively regulating PT growth through the transmitting tract (Liu et al., 2020).

Plantacyanin, which belongs to the phytocyanin family of blue copper proteins, has also been proposed to play an important role in PT guidance through the stigma. Plants overexpressing plantacyanin show reduced seed set, and PT guidance is disrupted. Pollen tubes growing on overexpression stigmas exhibit erratic growth patterns, making turns around papilla cells before growing towards the style (Dong et al., 2005).

Interestingly, a recent report showed a role for KATANIN (KTN1), a microtubule-severing enzyme that regulates cortical microtubule dynamics, guiding early PT growth in stigma papillae (Riglet et al., 2020). By studying *KTN1* mutants, the authors concluded that the KTN1-dependent cytoskeleton dynamics and the mechanical properties of the cell wall have a major role in guiding early pollen tube growth in stigma papillae (Riglet et al., 2020).

Additionally, the *Arabidopsis thaliana* pectin methylesterase homologous protein VANGUARD1 (VGD1) is secreted out of the PT, where it modifies the transmitting tract CW, facilitating PT penetration and growth (Jiang et al., 2005). Recently, it was also reported that the Tcb1-female barrier gene encodes a PME38 homolog. Pistils express Tcb1-f and modify the PT cell wall, which has been proposed as a mechanism for reproductive isolation in grasses (Lu et al., 2019).

Not only do chemical processes regulate PT growth and guidance through the transmitting tract, but physical mechanisms are also involved. During this journey through the female tissues, the CW plays an important role, providing mechanical support and also plasticity enough to permit not only growth but also a dynamic response to the plethora of chemical stimuli to which PTs respond. In this sense, the recently coined term “durotropic growth” refers to a mechano-sensitive growth behavior that contributes to PT guidance in plants with solid or semi-solid transmitting tracts (Reimann et al., 2020). Microtubules are also proposed to stabilize PT under tension, facilitating its growth (Hamant et al., 2019).

In the past few years, a great variety of molecules that participate in PT guidance have been described. A particularly important group is composed by synergid-secreted small cysteine-rich proteins (CRPs) named LURE. LURE proteins were first described in *Torenia fournieri* because of their ability to attract PTs of their own species *in vitro* (Okuda et al., 2009). After that, a cluster of five AtLUREs, which are specifically expressed in synergid cells and secreted towards the funicular surface, was identified in *A. thaliana* (Takeuchi and Higashiyama, 2012). Interestingly, it has been recently reported that knockout of the entire AtLURE1 gene family did not affect fertility but there was a simultaneous loss of function with four XIUQIU genes, which attract pollen tubes regardless of species, resulting in its reduction by approximately 20%. Thus, it has been proposed that species-specific AtLURE1s and non-species-specific XIUQIUs cooperate, contributing to proper guidance of pollen tubes and thus fertility (Zhong and Qu, 2019; Zhong et al., 2019). LURE1, a *Torenia concolor* ortholog, TcCRP1, has its function conserved (Kanaoka et al., 2011). In *Zea mays*, another CRP group called EGG APPARATUS 1 (ZmEA1) is exclusively expressed in the egg apparatus and is required for PT attraction (Márton et al., 2005). These molecular cues are perceived and transduced by multiple pollen tube-expressed receptor-like kinase (RLK) complexes (Johnson et al., 2019; Adhikari et al., 2020). Some examples include RECEPTOR-LIKE KINASE 6 (PRK6; Takeuchi and Higashiyama, 2016), and LOST IN POLLEN TUBE GUIDANCE 1 (LIP1) and 2 (LIP2; Liu et al., 2013). Among the molecular mechanisms underlying PT attraction by LUREs, it has been reported that several highly O-glycosylated arabinogalactan proteins (AGPs) are required in the PT to respond to LURE. The AGP AMOR, with a terminal 4-O-methyl-glucuronosyl residue, is sufficient to confer this competence to PTs (Dresselhaus and Coimbra, 2016). Interestingly, several AGPs have been identified along the transmitting tract: AGP1, AGP4 (JAGGER), AGP12, and AGP15 (Pereira et al., 2016a). While AGP1, AGP12, and AGP15 seem important to assure PT growth towards the ovule funiculus (Pereira et al., 2014), JAGGER appears to be involved in the signaling pathway that blocks the attraction of PT after fertilization, preventing polyspermy (Pereira et al., 2016b). Other transmitting tract AGPs are proposed to function as a nutrient source and to facilitate adhesion during PT growth (Pereira et al., 2016a).

## Cell Wall Remodeling and Integrity During Pollen Tube Growth

PT growth involves the regulation of CW remodeling and an active deposition of new CW. These processes dynamically modify the mechanical properties of the PT, which require a tight temporal and spatial control to promote fast PT elongation while ensuring CW integrity (Figure 2B).

CW synthesis during PT growth also involves multiple subcellular compartments. Most of the CW polymers are synthesized in the Golgi apparatus and secreted *via* Golgi-derived vesicles to fuse with the plasma membrane at the tip of the PT to sustain its growth (Dehors et al., 2019). These polymers include hemicelluloses, pectin motifs, and hydroxyproline-rich glycoproteins such as AGPs and extensins (EXTs). Indeed it has been recently shown that mutations in subunit 6 of the conserved oligomeric Golgi complex, COG6, result in aberrant PT growth. CW components are incorrectly deposited, highlighting the relevance of proper vesicle trafficking homeostasis PT growth (Rui et al., 2020). Other CW polymers, like cellulose and callose, are synthesized at the plasma membrane *via* CELLULOSE SYNTHASES (CESA) or CALLOSE SYNTHASES (CalS)/GLUCAN SYNTHASE-LIKE (GSL) complexes (Farrokhi et al., 2006).

One of the main CW components of angiosperms PTs is callose (Dardelle et al., 2010; Dehors et al., 2019). It has been demonstrated that callose is able to resist tension and compression stress, suggesting that it might have a mechanical role in growing PTs (Parre and Geitmann, 2005a). Callose is also periodically accumulated, forming callose plugs. As we have already mentioned, it maintains the cytoplasm near the growing tip, reducing the risk of damage and allowing the tube to grow long distances (Qin et al., 2012). This polymer is synthesized by proteins that belong to the CalS/GSL multigene family and usually form complexes. Although 12 GLS genes have been described in *A. thaliana*, only *AtCalS5* seems involved in PT growth and callose plug formation. Interestingly, knockout plants impaired in *AtCalS5* exhibit PTs that lack callose walls and plugs but are capable to fertilize ovules, questioning the role of callose in CW integrity (Nishikawa et al., 2005; Parre and Geitmann, 2005a). However, it is currently accepted that an inner callose wall and callose plugs were fundamental innovations associated with short-lived and fast-growing PTs that have triggered the ecological success of angiosperms. This polymer appears to be an evident evolutionary hinge at the sight of phylogenetic studies (Dehors et al., 2019).

Although cellulose is one of the most common polysaccharides in plant cell walls, it is weakly detectable in the PT shank (Dardelle et al., 2010). Cellulose is synthesized by the CESA complex and CELLULOSE SYNTHASE-LIKE D (CSLD) proteins (Farrokhi et al., 2006). *AtCSLD1* and *AtCSLD4* are highly expressed in mature pollen and PTs, where their encoding proteins were found in Golgi apparatus and in transport vesicles. PTs lacking CSLD1 and CSLD4 show PTs with reduced cellulose deposition and a significant disorganization of the PT wall layers. This disorganization caused the suppression of genetic transmission through the male gametophyte, indicating that cellulose synthesis is essential for maintaining PT integrity to reach the ovules (Wang et al., 2011). An insertional mutation in *AtCSLC6*, named as *tgd3*, also showed a strong inhibition of PT growth and reduced transmission through the male gametophyte (Boavida et al., 2009). Concordantly, when *Lilium auratum* and *Petunia hybrida* PTs are cultured in the presence of 2,6-dichlorobenzonitrile, a CESA inhibitor, the PTs frequently rupture or assume a bulbous shape (Anderson et al., 2002). All these data suggested that cellulose has an important role in maintaining CW integrity despite its low abundance in the PT. In addition and also supporting this role for cellulose in other species, CESA and CSLD proteins were found localized at the plasma membrane of *Nicotiana tabacum* PTs (Cai et al., 2011).

Pectin is the most abundant pollen CW polysaccharide and offers a variety of mechanical properties that are necessary for polarized growth (Ge et al., 2019a). Homogalacturonan (HG) consists of a linear a-1,4-linked galacturonic acid homopolymer, and it is assumed to be synthesized and methyl-esterified in Golgi and secreted as a highly methyl-esterified state. Therefore, it has been proposed that galacturonosyltransferase (GAUT) and pectin methyl-transferase act as a heterocomplex in HG synthesis (Wolf et al., 2009). Two GAUTs, GAUT13 and GAUT14, have been identified in *A. thaliana* as essentials for PT growth, as double mutants present defective elongation and swollen PTs (Wang et al., 2013). A polygalacturonate 4-α-galacturonosyltransferase has also been characterized from *Petunia axillaris* PTs (Akita et al., 2002), and a GAT-like gene, AtGATL4, was found to be expressed specifically in *Arabidopsis* PTs (Kong et al., 2011).

As we have mentioned before, HG can be substituted with methyl or acetyl groups, and that impacts on the mechanical properties of the CW. Weakly methylesterified HG strengthens the CW by forming a complex with HG chains and calcium. Removal of methyl and acetyl groups is coordinated by PMEs. PMEs have different modes of action depending on regulatory factors, such as pH, ion concentration, or existing state of methylesterification. In general, PMEs with acidic pH optima are thought to randomly demethylesterify pectins stimulating cleavages by polygalactunorases, resulting in CW loosening, whereas PMEs with an alkaline pH optima are thought to act consecutively or “in blocks,” favoring the formation of calcium-HG complexes stiffening the CW (Francis et al., 2006; Wolf et al., 2009). PTs are characterized by presenting highly esterified HG associated with the tip region and poorly esterified HG associated with the shank (Li et al., 1995; Jauh and Lord, 1996; Dardelle et al., 2010). This HG esterification pattern provides stiffness to the shank CW while maintaining tip plasticity, allowing for polar growth. Mutations in PME-encoding genes have shown the importance of their function in *A. thaliana*. For instance, *AtPME1* knockout plants exhibit curvy and irregular PTs (Tian et al., 2006). Similar results were obtained in the VGD1 and PME48 single mutants (Jiang et al., 2005; Leroux et al., 2015). In addition, *N. tabacum* mutants impaired in *NtPPME1* show PTs with significantly low growth rates (Li et al., 2002; Bosch and Hepler, 2006). *Z. mays* PMEs (ZmC5, ZmPme3, and ZmGa1P) have also been characterized and found to be involved in PT elongation (Zhang et al., 2019). PMEs can be classified into two types, type I and type II PMEs. Although both kinds share a catalytic PME domain at the C-terminal region, type I PMEs harbor an extra domain at the N-terminus, called the PRO region. This domain acts as a pectin methylesterase inhibitor (PMEI) domain, and its presence allows for PMEs to autoinhibit their methylesterase activity in specific situations. For instance, the PRO region of certain pollen-expressed type I PMEs might prevent PME activity during their transport in pectin-containing secretory vesicles (Micheli, 2001). This system relies on the activity of CW proteases that are able to release the pro-region from the enzymatic domain, thus allowing PME to catalyze de-esterification when needed (Bosch et al., 2005).

Additionally, PMEs can be regulated through the action of independent PMEIs (Röckel et al., 2008) that often form a stoichiometric 1:1 complex in which PMEIs block PMEs at their putative active site (Matteo et al., 2005). By inhibiting PME activity, they prevent excessive cross-linking of pectic homogalacturonan at the pollen tip CW, which then retains its ability to expand and grow (Woriedh et al., 2013; Paynel et al., 2014; Hocq et al., 2017). De-esterified homogalacturonan is found the in lateral regions of the PT, while esterified homogalacturonan is dominant at the PT apex (Bosch et al., 2005; Parre and Geitmann, 2005b). Evidence suggests that the localized secretion of specific PMEIs in the PT apex represents an efficient mechanism for the precise temporal and/or spatial regulation of PME activity in the PT. AtPMEI-YFP fusion proteins were exclusively localized to the *N. tabacum* PT apex despite YFP-tagged AtPPME1 being uniformly distributed throughout the entire pollen tube CW (Röckel et al., 2008). Additionally, PMEIs were also found to physically interact with PMEs and to abolish its endogenous activity *in vitro* (Röckel et al., 2008). Even lateral endocytic internalization was proposed as the mechanism responsible for the local distribution of PMEIs at the PT apex (Röckel et al., 2008). All these data suggest that the polarized accumulation of PMEIs at the PT apex contributes to CW integrity during PT growth by locally inhibiting PME activity (Figure 2B).

Demethylesterification of HG by PMEs at the PT tip is accompanied by proton release. The resultant local pH drop is thought to activate polygalactunorases and pectate lyases, which are also involved in the remodeling of the PT CW (Mollet et al., 2013). Their activity is also contributing to the loosening of the CW, facilitating tip growth. Accordingly, *Brassica campestris* double-mutant lacking the BcMF26a and BcMF26b polygalactunorases present PTs that cannot grow or stretch (Lyu et al., 2015). Although little information is available about pectate lyases-like proteins, antisense-RNA lines of *B. campestris* targeting BcPLL9 show PTs with growth arrest and uneven surface (Jiang et al., 2014).

Rhamnogalacturonan type II (RG-II) has the same homopolymer backbone as HG but is substituted with unusual sugars like aceric acid, apiose, 2-keto-3deoxy-D-lyxo-heptulosaric acid (Dha) and 2-keto-3-deoxy-D-mannooctulosonic acid (Kdo). RG-II forms dimers that are cross-linked by a borate di-ester bond between two apiosyl residues. Mutations in genes encoding for proteins involved in RG-II biosynthesis lead to abnormal PT growth phenotypes. Two of them are identified as MALE GAMETOPHYTE DEFECTIVE 2 and 4 (MGP2 and MGP4), corresponding to a sialyltransferase-like protein and a proposed xylosyltransferase, respectively (Deng et al., 2010; Liu et al., 2011). SIA2, another sialyltransferase-like protein, has been proposed to participate in the transference of Dha or Kdo to RG-II in *Arabidopsis* PTs (Dumont et al., 2014). Mutations in SIA2 result in PTs showing a swollen or dichotomous branching tip that was also much shorter compared to that of WT PTs (Dumont et al., 2014), indicating that RG-II plays an essential role in stabilizing the CW during PT growth.

As we have mentioned before, AGPs and extensins are tightly associated with the PT CW that have critical roles during polar growth. AGPs are found throughout the PT wall, with a stronger presence in the tip region, and are proposed to play a critical role in maintaining CW integrity (Dardelle et al., 2010; Lamport et al., 2018). Two classical AGPs (AtAGP6 and AtAGP11) and two AG-peptides (AtAGP23 and AtAGP40) are expressed in *Arabidopsis* PTs, and double-knockout mutant *agp6 agp11* presents severe PT growth phenotypes (Nguema-Ona et al., 2012; Lamport et al., 2018). Interestingly, based on their molecular structure and properties, AGPs are proposed to contribute to polar PT growth by constituting a source of cytosolic Ca^2+^ waves and serving as a pectic plasticizer (Lamport et al., 2018). It was also shown that disrupting AGP structure affects PT CWs by changing the distribution of other components, like cellulose, pectins, and callose, and hence affecting the CW mechanical properties (Leszczuk et al., 2019). All these data support the idea that AGPs are another brick in a complex wall whose purpose is to assure proper PT elongation while maintaining CW integrity.

Leucine-rich repeat extensins (LRXs) are EXTs that harbor a N-terminal leucine-rich repeat domain and a C-terminal extensin domain, with Ser-Pro (3-5) repetitive motifs likely involved in the interaction with CW components (Borassi et al., 2016). During reproductive growth, different combinations of *lrx* mutants show reduced fertility and decreased PG germination and PT growth when compared with WT plants (Fabrice et al., 2018; Sede et al., 2018; Wang et al., 2018). These defects were more severe in mutant lines that accumulated more *LRX* mutations, which suggests that LRXs are functionally redundant. PTs from *lrx* mutants show diverse PT defects. Some of them include PTs that are abnormally swelled or branched or that burst soon after emergence (Fabrice et al., 2018; Sede et al., 2018; Wang et al., 2018), PTs that grew intermittently (Fabrice et al., 2018) or present slow growth (Fabrice et al., 2018; Sede et al., 2018; Wang et al., 2018), PTs that display altered biophysical properties (Fabrice et al., 2018), aberrant discharge of vesicles at the apex (Fabrice et al., 2018), and PTs that present abnormalities in the abundance and distribution of ER/Golgi-synthesized CW components at the CW (Fabrice et al., 2018; Sede et al., 2018; Wang et al., 2018). This abnormal distribution of CW components was observed even when the expression profile of the genes involved in the biosynthesis and/or modification of CW polysaccharides was not altered (Wang et al., 2018). These results indicate the key role of LRXs in maintaining CW integrity and assuring proper CW assembly during PT growth. Sede et al. (2018) reported that in a *lrx9 lrx10 lrx11* triple mutant, callose is aberrantly accumulated at the point of PT emergence, while Wang et al. (2018) showed that the abundance of rhamnogalacturonan type I (RG-I), α-L-fucosylated XyG, cellulose, and callose was atypical at the PT apical and sub-apical regions in *lrx8 lrx10 lrx11* and *lrx9 lrx10 lrx11* triple mutants.

Fabrice et al. (2018) provided evidence that the levels of EXTs, AGPs, RG-I, unesterified and methyl-esterified HG, xyloglucan, and callose were all reduced in the CWs of *lrx8 lrx9*, *lrx8 lrx9 lrx11*, and *lrx8 lrx9 lrx10* mutant PTs. These defects were caused by deficient vesicle discharge and an incorrect integration of the newly synthesized material into the expanding CW, suggesting a role for LRXs in the coordination of these exocytosis steps in PT CW remodeling (Fabrice et al., 2018). Interestingly, *lrx* mutant PTs that grew on low-[Ca^2+^] conditions managed to partially overcome some of these phenotypes (Fabrice et al., 2018). As mentioned before, Ca^2+^ is a key component of PT growth, and these results suggest that LRXs may act as plasma membrane-CW signaling components of Ca^2+^-related processes (Fabrice et al., 2018).

## Pollen Tube Tip Rupture

**Figure 3:**
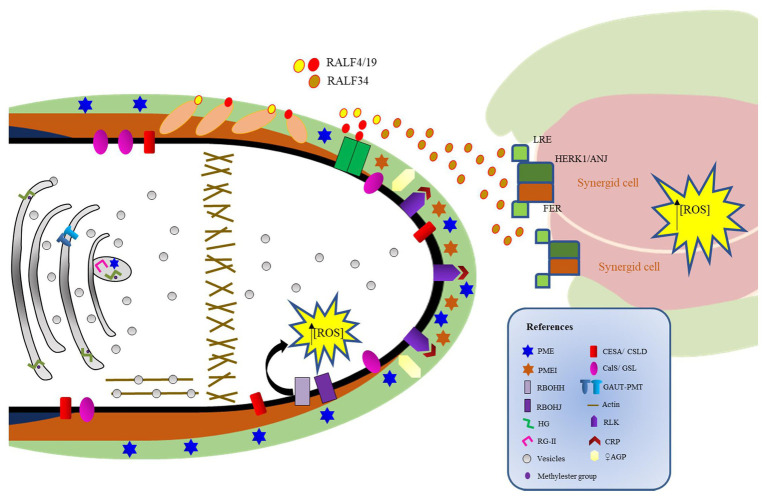
Regulation of pollen tube (PT) cell wall rupture. The receptor complex LLG2/3-BUPS1/2-ANX1/2 and its RALF4/19 ligands interact to sustain PT growth and prevent early PT rupture. RALF4/19 also interact with LRXs 8/9/10/11 to sustain PT growth. The LLG2/3-BUPS1/2-ANX1/2-RALF4/19 system is disturbed by ovule-derived RALF34, which competes with RALF4/19 for interaction with the receptor complex, promoting PT rupture and sperm cell delivery to the egg apparatus. In the embryo sac, FERONIA also contributes to PT rupture by elevating the concentration of reactive oxygen species and maintaining de-esterified pectin at the filiform apparatus.

PTs need to rupture as they reach the ovule in order to deliver the two sperm cells, a process that strongly depends on cell-cell communication mechanisms between the PT and the egg apparatus (Huck et al., 2003; Rotman et al., 2003). The PT burst requires a switch from PT integrity maintenance to rupture, a mechanism that involves multiple ligands and signaling cascades (Figure 3).

ANXUR1/2 (ANX1/2) are pollen-expressed RLKs that belong to the *Catharanthus roseus* RLK1-like subfamily (CrRLK1L) that are essential to regulate PT CW integrity. Concordantly, PTs from *anx1/anx2* mutants burst prematurely before arriving at the ovule (Boisson-Dernier et al., 2009; Miyazaki et al., 2009). ANX1/2 interact with two other CrRLK1L members, named BUPS1 and BUPS2, which are also required to maintain PT CW integrity during PT growth (Ge et al., 2017). Double mutants *bups1 bups2* showed PT that burst immediately after germination (Ge et al., 2017), similarly to the *anx1 anx2* mutants. Additionally, BUPS1/2 interact with two PT-expressed glycosylphosphatidylinositol-anchored proteins (GPI-APs), known as LORELEI-like-GPI-anchored protein 2 (LLG2) and LLG3 (Feng et al., 2019; Ge et al., 2019b). LLG2/3 act redundantly as co-receptors of the BUPS1/2-ANX1/2 receptor complex to sustain PT growth through the maternal tissues and to prevent early PT rupture (Feng et al., 2019; Ge et al., 2019b). This receptor complex binds to two secreted peptides known as rapid alkalinization factors (RALFs) 4/19. Double mutants for *RALF4/19* show phenotypes similar to the ones presented by mutants in *BUPS1/2*, *ANX1/2*, and *LLG2/3*, which also present PTs that burst prematurely (Boisson-Dernier et al., 2009; Miyazaki et al., 2009; Ge et al., 2017, 2019b; Mecchia et al., 2017). As RALF4/19 also interact with LRXs in the CW, it was proposed that RALF4/19 control PT growth and CW integrity in conjunction with LRXs (Mecchia et al., 2017). In this sense, it was recently proposed that RALF peptides could target LRX and CrRKL1L-LLGs signaling modules by two mutually exclusive binding events in the PT. Thus, RALF4/19 peptides can signal *via* the CrRLK1Ls-LLGs to control processes depending on the cytoplasmic RLK MARIS, or RALF4/19 may signal outside the cell through their interaction with LRXs in the CW (Moussu et al., 2020). As the affinity of RALFs for LLGs or LRXs changes depending on the pH and structural features of the ligands, it was suggested that cell wall pH and redox changes might alter the conformation states of RALF ligands, allowing the formation of different signaling complexes (Moussu et al., 2020).

In addition, Ge et al. (2017) hypothesized that the autocrine signaling mediated by RALF4/19 and the receptor complex ANX1/2-BUPS1/2 in the PT is inactivated by a ligand coming from the ovule upon PT arrival. RALF34 is an ovule-derived RALF that was able not only to interact with the ectodomains of both ANX1/2 and BUPS1/2 but also to compete with RALF4/19 for interaction with these CrRLK1Ls (Ge et al., 2017). Therefore, RALF34 could serve as an ovule-derived paracrine signal that disrupts the RALF4/19 PT autocrine signal, enabling PTs to rupture and deliver the sperm cells to the egg apparatus (Ge et al., 2017).

ANX1/2 are closely related to FERONIA (FER), a synergid-expressed CrRLK1L that also mediates male-female communication as the PT grows to reach the egg apparatus (Huck et al., 2003; Escobar-Restrepo et al., 2007). Plants defective in FER show invasive PTs, as they do not rupture and continue to grow inside the female gametophyte (Escobar-Restrepo et al., 2007). FER regulates the production of high levels of ROS at the micropylar region of the embryo sac, inducing PT rupture and sperm release (Duan et al., 2014). Remarkably, it was recently demonstrated that FER is essential for maintaining de-esterified pectin at the filiform apparatus, micropylar CW extensions at the site of PT entrance (Duan et al., 2020). Additionally, two CrRLK1L homologs, HERCULES RECEPTOR KINASE 1 (HERK 1) and ANJEA (ANJ), were recently described to promote PT growth arrest in the proximity of the synergid cells (Galindo-Trigo et al., 2020).

## Concluding Remarks

During its journey to reach the ovule, PT growth relies on massive CW deposition and fast CW remodeling that modifies its mechanical properties. These dynamic changes occur in tight coordination with female tissues, which play essential roles in PT nutrition and guidance, also regulating PT integrity. As a consequence, the PT cell wall results in a strong structure that supports cellular expansion but is flexible enough to respond to guidance cues. Although much research is needed to completely understand the mechanisms underlying these tightly regulated processes, the past few years have shed light on many aspects of PT integrity regulation. Although we know that PT CW integrity is sustained *via* an autocrine signaling pathway, its downstream effectors are yet to be identified. Particularly, those components that directly affect CW composition are still unknown. Characterization of these mechanisms will unravel the molecular basis of PT cell wall maintenance during growth and how its integrity, which is tightly preserved during its journey to the ovule, is suddenly lost upon reception.

## Author Contributions

MCs, NS, EZ, and GP took charge of conceptualization. GP, EZ, and AD were in charge of funding acquisition. GP and AD contributed to project administration. GP supervised the work. MCs, NS, and GP wrote the manuscript. AD, GL, NS, FM, MCs, MCi, and EZ reviewed and edited the manuscript. All authors contributed to the article and approved the submitted version.

### Conflict of Interest

The authors declare that the research was conducted in the absence of any commercial or financial relationships that could be construed as a potential conflict of interest.
